# Cessation of Smoking Trial in the Emergency Department: Long-Term Follow-up of a Randomized Controlled Trial

**DOI:** 10.1093/ntr/ntaf200

**Published:** 2025-09-29

**Authors:** Ian Pope, Zuzanna Halicka, Lucy Clark, Susan Stirling, Allan Clark, Caitlin Notley

**Affiliations:** Norwich Medical School, University of East Anglia, Norwich, UK; Norwich Clinical Trials Unit, University of East Anglia, Norwich, UK; Norwich Clinical Trials Unit, University of East Anglia, Norwich, UK; Norwich Clinical Trials Unit, University of East Anglia, Norwich, UK; Norwich Clinical Trials Unit, University of East Anglia, Norwich, UK; Lifespan Health Research Centre, Norwich Medical School, University of East Anglia, Norwich, UK

## Abstract

**Introduction:**

Emergency departments (EDs) offer a valuable opportunity to deliver smoking cessation interventions. Long-term abstinence confers the maximum health benefits.

**Methods:**

Adults attending UK EDs who currently smoked were randomized to an intervention (brief advice, e-cigarette, and referral to local stop smoking services) or control (contact details for local stop smoking services). Participants were followed up at 1, 3, and 6 months as part of the main trial. Participants who consented to long-term follow-up were also contacted at approximately 18 months post randomization. For an “all participants” analysis, those who did not consent to long-term follow-up had their smoking status set at the value of the 6-month outcome. Those who did not respond were assumed to be smoking.

**Results:**

Long-term follow-up occurred between 14 and 22 months, mean = 18 months. Long-term follow-up for those who consented to this was 35% in the intervention group (*n* = 145) and 34% in the control group (*n* = 143). For those who consented to long-term follow-up self-reported 7-day abstinence at 18 months was 12.8% in the intervention group (*n* = 53) and 8.33% in the control group (*n* = 35) (RR = 1.56, 95% CI = 1.04%−2.32%, *p* = .031). For all participants self-reported 7-day abstinence at long-term follow-up was 13.8% in the intervention group (*n* = 67) and 8.6% in the control group (*n* = 42) (RR = 1.61, 95% CI = 1.12%−2.31%, *p* = .010).

**Conclusions:**

Adults who smoke attending the ED who received a smoking cessation intervention were significantly more likely to report abstinence 18 months after randomization.

**Implications:**

Emergency Departments should be considered as a location for smoking cessation interventions in order to increase long-term abstinence.

## Introduction

The emergency department (ED) offers a promising location to administer smoking cessation interventions given the ability to access large numbers of people experiencing complex health inequalities and the fact that it represents a “teachable moment” when people are thinking about their health.^1^ Previous studies have shown efficacy for ED-based smoking cessation interventions at shorter follow-up periods; however, evidence is scarce for longer term follow-up.^2^

Given the risk of relapse after smoking cessation, guidelines advocate the use of a 12-month follow-up period to assess sustained abstinence and provide confidence that it will continue long-term and that the health benefit will be maximized.^3^^,^^4^

Five previous ED-based randomized trials have undertaken 12-month follow-up following a smoking cessation intervention.^5-9^ Three of these provided behavioral support alone, with two offering nicotine replacement therapy. Only one found a statistically significant difference in smoking rates at 12 months. No ED-based trials have completed longer than 12-month follow-up. Of the three trials that reported 6- and 12-month cessation data the relapse rates in the intervention groups were between 0% and 2%.^5-7^

In this trial, we aimed to test the long-term real-world effectiveness of an ED-based brief smoking cessation intervention in comparison with usual care, by comparing self-reported smoking abstinence at around 18-month follow-up between trial groups.

## Methods

### Trial Design

The Cessation of Smoking Trial in the Emergency Department (COSTED) is a two-arm, multicentre, randomized controlled trial conducted in six UK NHS EDs.^10^ Further details on the trial design can be found in the published protocol and trial outcome paper, reporting 6-month biochemically confirmed abstinence results as the primary outcome.^10^^,^^11^

### Participants

Adults (aged 18 years or older) who smoked tobacco daily and were either seeking medical treatment in the ED or accompanying someone seeking medical treatment were screened for eligibility while waiting to be seen or after discharge.

Where the person accompanying an included patient met the inclusion criteria and wished to participate, they were enrolled in a similar way to the patients and assigned to the same treatment group as the patient they accompanied. They were not contacted for long-term follow-up so are not included in this analysis.

### Randomization

Participants were randomly assigned to either the intervention or control group using an automated web-based service from the Norwich Clinical Trials Unit.^11^ The intervention required active participation, therefore, it was not possible to blind participants or those delivering the intervention.

### Interventions

Participants randomized to the intervention group received a personalized smoking cessation intervention in the ED from a trained smoking cessation advisor who provided brief smoking cessation advice (up to 15 min), provision of a nicotine e-cigarette and advice on how to use it (up to 15 min) and referral to local smoking cessation services.^12^ The control group received written information about local-stop smoking services but were not referred.

### Long-Term Follow-Up Group

As part of the trial consent process and prior to randomization, participants were asked if they agreed to be contacted about future ethically approved research studies. Those who consented were contacted at or near to 18-months post randomization. These participants form the “Long term follow-up group.”

### All Participants Group

To allow analysis of the whole trial patient population, for the analysis of the “all participants group,” patient participants who did not consent to long-term-follow-up had their smoking status imputed, assuming it had not changed since 6-month follow-up, using a “last observation carried forward” approach. Those who did not respond at 6 months were recorded as smoking as per the Russel Standard.^3^

### Follow-up Procedures

Follow-up data to determine smoking status were collected at four intervals post-randomization—1, 3, 6, and around 18 months.

At 1, 3, and 6 months, participants received up to five separate contacts (text, phone, email, or post) before being classified as lost to follow-up.

Participants who completed the 6-month questionnaire received a £30 shopping voucher to show appreciation for their time.^11^

The long-term follow-up group were sent an automated text message, email, or paper form (with a stamped return envelope), to assess self-reported 7-day abstinence. Participants were sent an automated reminder to respond 3 days after the initial attempt or posted a second paper form. No further follow-up attempts were made.

Both initial and follow-up reminders were sent at the same time to all participants in the long-term follow-up group, regardless of when they were randomized, meaning the time between randomization and follow-up varied between 13 and 22 months.

### Outcomes

We measured self-reported 7-day abstinence at 1, 3, 6, and around 18-months by asking participants: “Have you smoked a tobacco cigarette in the past 7 days?”. Participants were classified as abstinent from smoking if they answered “no.” If a participant did not respond they were assumed to be smoking, according to the Russel Standard.^3^

Other measures collected on recruitment were demographics (gender, age, ethnicity, deprivation, and education level), median number of cigarettes smoked per day, mean motivation to quit smoking (measured using the motivation to quit smoking scale^13^), mean age started smoking, mean Fagerström test for nicotine dependence score, use of nicotine replacement therapy, or e-cigarettes in the last 3 months and whether they lived with someone who smokes.

### Statistical Analysis

In order to include all participants in “all participant group” analysis, the following assumptions were made:

- Individuals who did not provide consent to be contacted had the outcome set at the value of the 6-month self-reported outcome along with the time since randomization set to 6 months;
- Individuals who did provide consent to be contacted but did not respond are assumed to have returned to smoking along with the time since randomization set to 12 months;
- Individuals who did provide consent to be contacted and did respond had their response in the dataset set to the response they provided and the time since randomization set to the number of whole months since randomization.

This outcome was compared between treatment groups using a log-binomial regression adjusting for site and time since randomization in months. This allowed the estimation of the relative risk of abstinence between the two treatment groups. An additional model using a binomial regression with an identity link, adjusting for site and time since randomization in months, was also used. This allows the estimation of the risk difference of abstinence between the two treatment groups. In order to assess the assumption of the missing data, the following additional analyses were conducted for the long-term outcome: (1) using the observed responses at 18 months in this model we did not adjust for time since randomization; this assesses the missingness assuming missing at random; (2) using the observed responses at 18 months in the model, we adjusted for factors that were predictive of missingness, this assess the missingness assuming missing at random; (3) using pattern-mixture model of White et al. ^14^ where the informative missing odds ratio (IMOR) ranged from the most extreme missing not at random assumption of IMOR = 0 (assuming missing = smoking) to missing at random IMOR = 1 for this model, it was only possible to estimate the odds ratio rather than the risk difference of relative risk. The results are presented separately for those who consented to long-term follow-up and for those who did not. The rates for other time-points in the COSTED trial are given for the ease of comparison.

### Patient and Public Involvement

The trial was guided by patient and public involvement (PPI) from the beginning with consultations in EDs to evaluate the approach to smoking cessation and by the support of PPI volunteers who helped with trial setup, reviewing materials, suggesting language improvements and choosing the e-cigarette for the intervention.^11^

## Results

**Figure 1:**
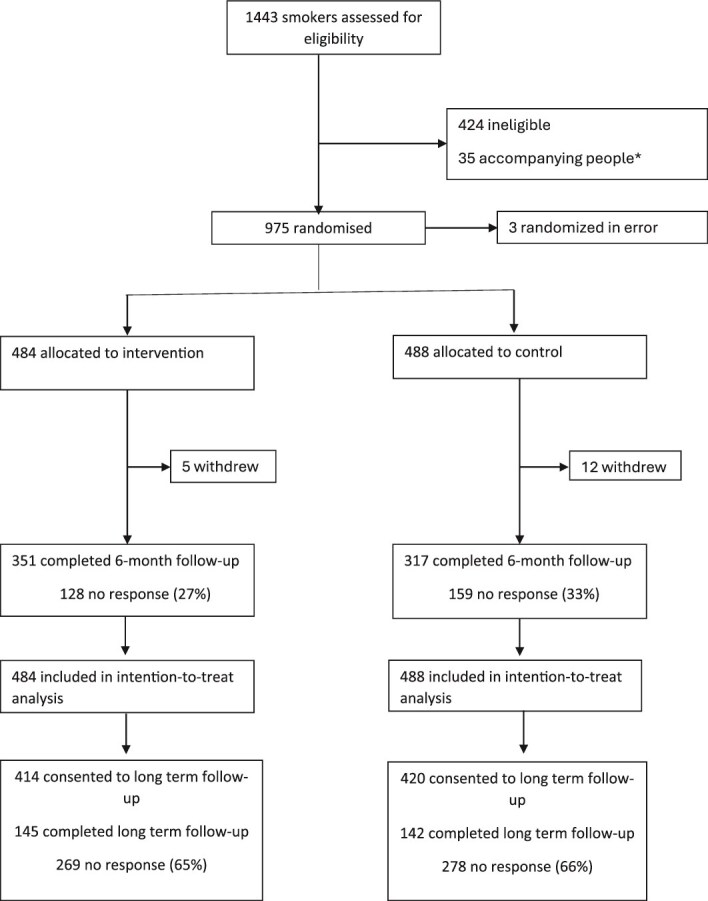
Trial profile.

A total of 972 patient participants were randomized between January and August 2022 (484 intervention, 488 control). Of the participants 85.5% (*n* = 834, 414 intervention, 420 control) consented to long-term follow-up. A total of 17 withdrew (5 from intervention and 12 from control) during trial follow-up. Figure 1 shows the flow of participants.

Baseline characteristics are shown in Table 1, with a comparison between those who consented to long-term follow-up and those who did not. The groups are broadly similar; however, more women than men consented, a greater proportion of White ethnicity participants consented, with less consent from other ethnic groups, and more of those recruited in Norwich and Leicester consented. The motivation to quit score of 4 correlates to a response “I REALLY want to stop smoking but I don’t know when I will.”

**Table 1:** Baseline Characteristics by Consent Status for Long-Term Follow-up

		Did not consent (*n* = 138)	Consented (*n* = 834)	Total (*n* = 972)
Randomization group	Control	68 (49.3%)	420 (50.4%)	488 (50.2%)
	Intervention	70 (50.7%)	414 (49.6%)	484 (49.8%)
Gender	Male	100 (72.5%)	503 (60.3%)	603 (62.0%)
	Female	38 (27.5%)	331 (39.7%)	369 (38.0%)
Mean age (years) (SD)		42.92 (15.39)	40.10 (13.30)	40.50 (13.65)
Ethnic origin	White British	74 (53.6%)	629 (75.4%)	703 (72.3%)
	White – Other	22 (15.9%)	100 (12.0%)	122 (12.6%)
	Black	14 (10.1%)	43 (5.2%)	57 (5.9%)
	South Asian	21 (15.2%)	43 (5.2%)	64 (6.6%)
	Other	7 (5.1%)	17 (2.0%)	24 (2.5%)
	Refused / missing	0 (0.0%)	2 (0.2%)	2 (0.2%)
Mean deprivation decile (SD)		4.12 (2.43)	4.48 (2.62)	4.42 (2.59)
Employment status	Employed	85 (61.6%)	511 (61.3%)	596 (61.3%)
	Unemployed	15 (10.9%)	81 (9.7%)	96 (9.9%)
	Unable to work due to sickness or disability	23 (16.7%)	153 (18.3%)	176 (18.1%)
	Carer, retired or student	15 (10.9%)	87 (10.4%)	102 (10.5%)
	Other	0 (0.0%)	2 (0.2%)	2 (0.2%)
Median number of cigarettes smoked per day (IQR)		15 (10 - 20)	15 (10 - 20)	15 (10–20)
Mean motivation to quit score (SD)		4.07 (1.56)	4.14 (1.61)	4.13 (1.60)
Mean age started smoking (SD)		15.92 (4.10)	15.80 (4.71)	15.82 (4.63)
Mean Fagerström test for nicotine dependence score (SD)		4.86 (2.35)	4.90 (2.30)	4.89 (2.31)
Use of nicotine replacement therapy in last 3 months		14 (10.1%)	74 (8.9%)	88 (9.1%)
Use of e-cigarettes in the last 3 months	Not used	105 (76.1%)	617 (74.0%)	722 (74.3%)
	Once a month or less	15 (10.9%)	79 (9.5%)	94 (9.7%)
	On 2−4 days a month	4 (2.9%)	52 (6.2%)	56 (5.8%)
	On 2−3 days a week	10 (7.2%)	39 (4.7%)	49 (5.0%)
	On 5−6 days a week	4 (2.9%)	47 (5.6%)	51 (5.3%)
	Daily	0	0	0
Lives with other smoker(s)		50 (36.2%)	349 (41.8%)	399 41.1%)
Recruitment by site	Norwich	12 (8.7%)	388 (46.5%)	400 (41.2%)
	London	58 (42.0%)	110 (13.2%)	168 (17.3%)
	Homerton	18 (13.0%)	89 (10.7%)	107 (11.0%)
	Leicester	10 (7.2%)	140 (16.8%)	150 (15.4%)
	Edinburgh	14 (10.1%)	86 (10.3%)	100 (10.3%)
	Addenbrookes	26 (18.8%)	21 (2.5%)	47 (4.8%)

Long-term follow-up was completed by 34.4% of participants in the long-term follow-up group (*n* = 287, 145 intervention, 142 control) (Figure 1). This compares to a follow-up rate at 6 months of 68.7% at 6 months (*n* = 668, 351 intervention, 317 control).

Duration of follow-up for those who consented to long-term follow-up ranged from 13.8 to 22.0 months (range = 419−668 days, mean = 536 days, approximately 17.6 months).

For those who consented to long-term follow-up, the self-reported 7-day abstinence at long-term follow-up was 12.80% in the intervention group (*n* = 53) and 8.33% in the control group (*n* = 35) (RR = 1.56, 95% CI = 1.04%−2.32%, *p* = .031) (Table 2).

**Table 2:** Abstinence Rates at Different Time Points for Those Who Consented to Long-Term Follow-up

	Intervention (*n* = 414)	Control (*n* = 420)	Absolute difference (95% CI)	*p*-value	Relative risk (95% CI)	*p*-value
Self-reported 7 day abstinence at 1 month	85 (20.5%)	42 (10.0%)	10.1 (5.3, 14.9)	<.001	2.03 (1.44, 2.85)	<.001
Self-reported 7 day abstinence at 3 months	100 (24.2%)	50 (11.9%)	11.9 (6.7, 17.1)	<.001	2.02 (1.48, 2.75)	<.001
Self-reported 7 day abstinence at 6 months	100 (24.2%)	57 (13.6%)	10.5 (5.3, 15.8)	<.001	1.75 (1.30, 2.35)	<.001
Self-reported 7 day abstinence at around 18 months^a^	53 (12.80%)	35 (8.33%)	Not converged		1.56 (1.04, 2.32)	.031
Self-reported 7 day abstinence at around 18 months^a^^,^^b^	53 (12.80%)	35 (8.33%)	4.6 (0.58, 8.7)	.025	1.55 (1.03, 2.34)	.034

a Adjusted for time since randomization.

b Omitting site.

The relative risk of abstinence was relatively consistent (2.03 at 1 month, 2.02 at 3 months, 1.75 at 6 months, and 1.56 at 18 months); however, the proportion of the intervention group reporting abstinence was higher at earlier time points (20.5% in 1 month, 24.2% at 3 months, 24.2% at 6 months, and 12.8% at 18 months).

For the “all participants” group (where those who responded at 6-months, but who did not consent at long-term follow-up had their smoking status set as the 6-month value) self-reported 7-day abstinence at long-term follow-up was 13.8% in the intervention group (*n* = 67) and 8.6% in the control group (*n* = 42) (RR = 1.61, 95% CI = 1.12%−2.31%, *p* = .010) (Table 3). This effect between groups remained significant in all of the sensitivity models for missing data. The estimated difference was highest in the observed only analysis (OR = 1.75, 95% CI = 1.05%−2.94%) and smallest for the standard assumption of missing data is equivalent to smoking (OR = 1.60, 95% CI = 1.02%, 2.51%) (Supplementary tables 1 and 2).

**Table 3:** Abstinence Rates at Different Time Points for All Participants

	Intervention (*n* = 484)	Control (*n* = 488)	Absolute difference (95% CI)	*p*-value	Relative risk (95% CI)	*p*-value
Self-reported 7 day abstinence at 1 month	94 (19.4%)	49 (10.0%)	9.0 (4.9−13.7)^**1**^	<.0001	1.92 (1.39−2.64)	<.0001
Self-reported 7 day abstinence at 3 months	113 (23.3%)	58 (11.9%)	11.3 (6.6−16.1)	<.0001	1.97 (1.47−2.63)	<.0001
Self-reported 7 day abstinence at 6 months	113 (23.3%)	63 (12.9%)	10.6 (5.86, 15.41)	<.0001	1.80 (1.36−2.38)	<.0001
Self-reported 7 day abstinence at around 18 months^a^	67 (13.8%)	42 (8.6%)	5.2 (1.3, 9.0)	.008	1.61 (1.12−2.31)	.010

a Adjusted for time since randomization (months).

 Full details of the 6-month results including biochemically verified smoking status and use of e-cigarettes can be found in the published results^11^ and details of participant use of smoking cessation services can be found in a mixed methods exploration of this topic.^15^

## Discussion

### Principal Findings

In this trial, adults attending the ED who received a brief opportunistic smoking cessation intervention were significantly more likely to report 7-day abstinence at or near to 18 months later compared to those receiving usual care.

### Comparison with Previous Studies

Previous ED smoking cessation trials have generally reported modest and short-lived effects. In comparison, this trial has demonstrated the largest intervention effect size in terms of absolute difference in smoking status outcome between comparison groups of any ED-based smoking cessation trial at or beyond a 12-month follow-up timepoint. The relative risk of abstinence at or beyond 12 months was also the highest of any previous trial indicating greater abstinence rates in the intervention arm compared to control. It is also only the second to find a significant difference between the groups at or beyond 12 months.

Potential causes of the higher quit rates are the use of e-cigarettes, which have been demonstrated to be more effective than nicotine replacement therapy^16^ and the immediate provision of support and pharmacotherapy, rather than it being delivered after the patient has been discharged, which could result in disengagement.

The fact that relative risk of abstinence remained relatively stable between 1 and 18 months is a notable finding, particularly given the very brief nature of the intervention (30 min of advice and 2 weeks of supplies) and the very low rates of engagement with stop-smoking services after referral (around 3.2% in the intervention group).^15^ This suggests the impact of the intervention persists without any further input.

In contrast to previous studies,^5-9^ the proportion of participants reporting abstinence fell between 6 and 18 months; however, this may be due to reduced follow-up rates at 18 months as a result of fewer contact attempts.

This was the first ED trial to include e-cigarettes as part of a smoking cessation intervention. The relative risk of abstinence at all follow-up points was broadly in keeping with that of other trials comparing nicotine e-cigarettes with behavioral support alone/ no support.^16^ Providing evidence for the effectiveness of e-cigarettes for achieving long-term abstinence.

### Strengths and Limitations

The strengths of this study are the relatively large sample size, the diversity of recruitment centers, which enhances generalizability, and the unusually long follow-up. A further strength was the low-intensity nature of the intervention, therefore making implementation easier.

The weaknesses are that this follow-up was not initially planned at trial launch and was therefore not described in the initial statistical analysis plan. Due to practicality, all participants were followed up at the same time; therefore, exact follow-up timepoints varied within a range of 13.8−22.0 months. The relatively low follow-up rates of those who consented to be contacted (although equal between the groups) and the lack of biochemical verification are further limitations. The lower response rate to the 18-month follow-up compared to other time points is likely due to the unplanned nature of this follow-up, and therefore the more limited number of attempts to contact people (two vs. up to five).

## Conclusion

This trial demonstrates that a brief intervention that was effective for smoking cessation at 6 months is also effective at supporting long-term smoking abstinence, even without further intervention or support.

## Supplementary Material

COSTED_long_term_follow-up_supplementary_materials_ntaf200

## Data Availability

The protocol, consent form, statistical analysis plan, medical ethics committee approvals, training materials, and other relevant study materials are available online at https://osf.io/8hbne/. Deidentified participant data will be made available upon reasonable request.
