# Supplementary material for: Cessation of Smoking Trial in the Emergency Department: Long-Term Follow-up of a Randomized Controlled Trial
Source: Nicotine Tob Res. 2025 Sep 29;28(4):686–91. doi: 10.1093/ntr/ntaf200 (PMC13008581; doi:10.1093/ntr/ntaf200)
Supplement: COSTED_long_term_follow-up_supplementary_materials_ntaf200 [file costed_long_term_follow-up_supplementary_materials_ntaf200.docx]

Supplementary table 1- Results of sensitivity analysis

|  | OR | p-value | RR | p-value | RD | p-value |
| --- | --- | --- | --- | --- | --- | --- |
| Primary analysis  (LOCF)^1^ | 1.62 (1.03,2.55) | 0.037 | 1.52 (1.02,2.28) | 0.042 | 4.6 (0.5,8.7) | 0.028 |
| Analysis assuming no-consent or missing = smoking^2^ | 1.62 (1.03,2.55) | 0.037 | 1.52 (1.02,2.28) | 0.042 | 4.6 (0.5,8.7) | 0.028 |
| Analysis based on observed data only (MCAR)^3^ | 1.75 (1.05,2.94) | 0.032 | 1.45 (1.01,2.07) | 0.041 | 12.3% (1.8-22.8) | 0.022 |
| Analysis based on observed data only adjusting for predictive factors of missingness (MAR)^4^ | 1.88 (1.09,3.24) | 0.023 | Did not converge |  | Did not converge |  |
| MNAR (IMOR)^5^ |  |  |  |  |  |  |
| 0 | 1.62 (1.02,2.56) |  |  |  |  |  |
| 0.1 | 1.69 (1.05,2.72) |  |  |  |  |  |
| 0.2 | 1.73 (1.06,2.81) |  |  |  |  |  |
| 0.3 | 1.75 (1.06,2.81) |  |  |  |  |  |
| 0.4 | 1.76 (1.06,2.91) |  |  |  |  |  |
| 0.5 | 1.77 (1.06,2.93) |  |  |  |  |  |
| 0.6 | 1.77 (1.06,2.94) |  |  |  |  |  |
| 0.7 | 1.77 (1.06,2.95) |  |  |  |  |  |
| 0.8 | 1.77 (1.06,2.95) |  |  |  |  |  |
| 0.9 | 1.76 (1.05,2.95) |  |  |  |  |  |
| 1.0 | 1.75 (1.05,2.94) |  |  |  |  |  |

^1^ Adjusting for site

^2^Adjusting for site, age, gender, patient/accompanying person, white British vs other, deprivation index, time since randomisation, if the talked to GP about quitting.

^3^ Adjusting for site, with auxillary variables in the imputation model of age, gender, patient/accompanying person, white British vs other, deprivation index, time since randomisation, if the talked to GP about quitting. Site was not included due to collinearity.

Supplementary table 2- characteristics by response status

|  | | | No Long-term response  (n = 138) | Long-term response  (n = 834) | p-value |
| --- | --- | --- | --- | --- | --- |
| Randomisation Group | Control | | 346 (50.5%) | 142 (49.5%) | 0.77 |
|  | Intervention | | 339 (49.5%) | 145 (50.5%) |  |
| Gender | Male | | 459 (67.0%) | 144 (50.2%) | <0.001 |
|  | Female | | 226 (33.0%) | 143 (49.8%) |  |
| Mean age (years) (SD) | | | 39.40 (13.27) | 43.11 (14.18) | <0.001 |
| Ethnic origin | White British | | 476 (69.5%) | 227 (79.1%) | 0.005 |
|  | White – Other | | 88 (12.8%) | 34 (11.8%) |  |
|  | Black | | 43 (6.3%) | 14 (4.9%) |  |
|  | South Asian | | 58 (8.5%) | 6 (2.1%) |  |
|  | Other | | 18 (2.6%) | 6 (2.1%) |  |
|  | Refused / missing | | 2 (0.3%) | 0 (0.0%) |  |
| Mean deprivation decile (SD) | | | 4.31 (2.56) | 4.70 (2.65) | 0.034 |
| Employment status | Employed | | 419 (61.2%) | 177 (61.7%) | 0.39 |
|  | Unemployed | | 69 (10.1%) | 27 (9.4%) |  |
|  | Unable to work due to sickness or disability | | 130 (19.0%) | 46 (16.0%) |  |
|  | Carer, retired or student | | 65 (9.5%) | 37 (12.9%) |  |
|  | Other | | 2 (0.3%) | 0 (0.0%) |  |
| Median number of cigarettes smoked per day (IQR) | | | 15.00 (10.00, 20.00) | 15.00 (10.00, 20.00) | 0.074 |
| Mean motivation to quit score (SD) | | | 4.15 (1.58) | 4.08 (1.66) | 0.54 |
| Mean age started smoking (SD) | | | 15.80 (4.46) | 15.85 (5.03) | 0.88 |
| Mean Fagerström test for nicotine dependence score (SD) | | | 4.84 (2.30) | 5.02 (2.31) | 0.26 |
| Use of nicotine replacement therapy in last 3 months | | | 68 (9.9%) | 20 (7.0%) | 0.14 |
| Use of e-cigarettes in the last 3 months | | Not used | 505 (73.7%) | 217 (75.6%) | 0.33 |
|  | | Once a month or less | 71 (10.4%) | 23 (8.0%) |  |
|  | | On 2-4 days a month | 36 (5.3%) | 20 (7.0%) |  |
|  | | On 2-3 days a week | 39 (5.7%) | 10 (3.5%) |  |
|  | | On 5-6 days a week | 34 (5.0%) | 17 (5.9%) |  |
|  | | Daily |  |  |  |
| Lives with other smoker(s) | | | 280 (40.9%) | 119 (41.5%) | 0.870 |
| Recruitment by site | Norwich | | 261 (38.1%) | 139 (48.4%) | <0.001 |
|  | London | | 137 (20.0%) | 31 (10.8%) |  |
|  | Homerton | | 72 (10.5%) | 35 (12.2%) |  |
|  | Leicester | | 117 (17.1%) | 33 (11.5%) |  |
|  | Edinburgh | | 58 (8.5%) | 42 (14.6%) |  |
|  | Addenbrookes | | 40 (5.8%) | 7 (2.4%) |  |
